# Impacts of MDR/XDR-TB on the global tuberculosis epidemic: Challenges and opportunities

**DOI:** 10.1016/j.crmicr.2024.100295

**Published:** 2024-10-19

**Authors:** Kai Ling Chin, Luis Anibarro, Zi Yuan Chang, Praneetha Palasuberniam, Zainal Arifin Mustapha, Maria E. Sarmiento, Armando Acosta

**Affiliations:** a Faculty of Medicine and Health Sciences, Universiti Malaysia Sabah, Kota Kinabalu, Sabah, Malaysia; b Borneo Medical and Health Research Centre, Faculty of Medicine and Health Sciences, Universiti Malaysia Sabah, Kota Kinabalu, Sabah, Malaysia; c Tuberculosis Unit, Infectious Diseases, and Internal Medicine Department, Complexo Hospitalario Universitario de Pontevedra, Pontevedra, Spain; d Immunology Research Group, Galicia Sur Health Research Institute (IIS-GS), Vigo, Spain; e Formerly School of Health Sciences, Universiti Sains Malaysia, Kubang Kerian, Kelantan, Malaysia. Independent Researcher

**Keywords:** MDR-TB, XDR-TB, diagnosis, Treatment, Vaccine

## Abstract

Tuberculosis (TB) is the world's second-deadliest infectious disease. Despite the availability of drugs to cure TB, control of TB is hampered by the emergence of multidrug-resistant tuberculosis (MDR-TB) and extensively drug-resistant tuberculosis (XDR-TB). The presence of MDR/XDR-TB is alarming due to the low detection rate, high treatment failure, and high mortality. The increasing cases of MDR/XDR-TB are mainly due to the limitations in the diagnostic tests to detect the drug susceptibility of the pathogen, which contribute to the spread of the disease through close contacts. Moreover, inconsistent drug therapy or unsuitable drug regimens could also lead to the subsequent development of drug resistance. The close contacts of an index MDR/XDR-TB patient are at increased risk of developing MDR/XDR-TB. Also, the BCG vaccine may exhibit varying protective effects due to BCG strain diversification, host immune status, exposure to environmental non-tuberculous mycobacteria (NTM), and differences in *Mycobacterium tuberculosis* (Mtb) subspecies infection, as in the case of sub-optimal protection in the case of Beijing family genotypes of Mtb. This review provides an overview of the current state of drug-resistant tuberculosis (DR-TB) within the context of the global TB pandemic, with a focus on diagnosis, treatment, and the potential impact of BCG vaccination. It highlights the limitations of current approaches and aims to identify opportunities for improving TB control strategies.

## Highlights

• Low detection rates and high treatment failure of DR-TB hinder TB control.
• Limitations in diagnostics of DR-TB and the importance of efflux pumps.
• Unsuitable drug regimens for DR-TB and the importance of alternative treatments.
• Less protective effect of BCG vaccine and new vaccines in clinical trials.

## Graphical abstract

**Figure:**
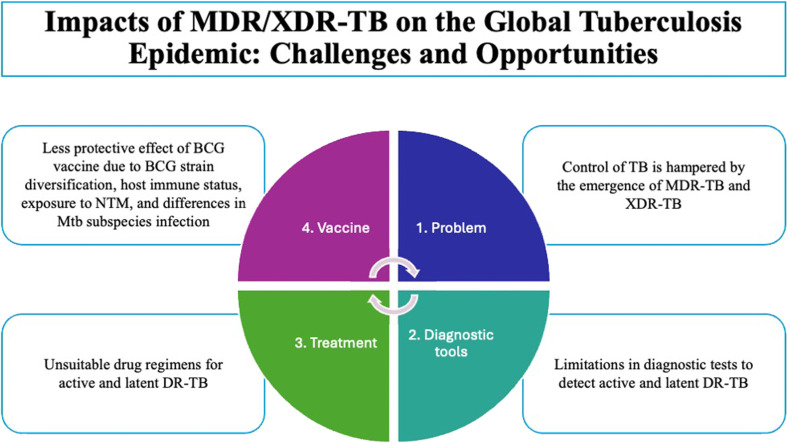

## Introduction

Tuberculosis (TB) is one of the deadliest infectious diseases, only surpassed by the coronavirus disease (COVID-19) since 2020. The COVID-19 pandemic is having an ominous impact on TB control due to restricted access to disrupted TB services, reversing the advances achieved in the last years. Due to COVID-19, TB deaths increased, for the first time since 2015 (Ntoumi et al., 2022).

Globally, nearly one-fourth of the world's population is infected by *Mycobacterium tuberculosis* (Mtb) [also known as tuberculosis infection (TBI)] and about 5–10% of the infected persons will develop active TB (ATB) at some time in their lives, especially during the first two years of infection (Reichler et al., 2018). Worldwide, an estimated 10.6 million people developed TB disease in 2022, causing 1.3 million deaths (WHO, 2023). The burden of multidrug-resistant tuberculosis [MDR-TB, resistance to first-line anti-TB drugs, isoniazid (INH) and rifampicin (RIF)] or RIF-resistant tuberculosis (RR-TB) has been estimated in 410,000 cases. Among those patients who were microbiologically tested, 27,075 had pre-extensively drug-resistant TB (pre-XDR-TB) cases [a form of MDR-TB with additional resistance to any fluoroquinolone (FQ)] or XDR-TB (a form of MDR-TB resistant to any FQ and at least one of the Group-A drugs [currently any FQ, bedaquiline (BDQ), and linezolid (LZD)]. Globally, MDR-TB is present in 3.3% of patients with newly diagnosed TB, and 17% among those patients who have a history of previous TB treatment. The highest proportion of MDR-TB are found in Russia and several countries in Asia and East Europe (WHO, 2023).

Despite the developments in diagnosis and treatment for TB, only 73% of bacteriologically confirmed pulmonary TB (PTB) were tested for RIF resistance and one in three of the people who develop MDR/RR-TB was treated in 2022, suggesting that the MDR-TB cases is still under-reported and portray hazards to the community (WHO, 2023). MDR-TB can be classified into “resistance in new patients” who acquire the contagious disease from an MDR-TB index patient or “resistance in previously treated patients” who develop MDR-TB due to improper prescriptions in susceptible patients, incomplete full course drug treatment, or suboptimal adherence to TB treatment (Tiberi et al., 2019). To cope with the increasing burden of drug-resistant tuberculosis (DR-TB), particularly MDR/XDR-TB, is of paramount importance to detect the active DR-TB cases and all the close contacts (Zhou et al., 2024).

This review aims to present a comprehensive overview of DR-TB, with an emphasis on diagnosis, treatment, and the potential role of BCG vaccination. It examines the shortcomings of current methods and highlights opportunities to enhance TB control strategies.

## Diagnosis of drug-resistant tuberculosis

### 2.1 Phenotypic characterization of DR-TB

Universal drug-susceptibility testing (DST) is the gold standard for detecting DR-TB cases. It is a phenotypic-based method to detect the growth of Mtb under the presence of antibiotics either on solid or liquid media (WHO, 2014). Mtb is a slow-grower, which takes 3–8 weeks to grow on solid media and 1–3 weeks in liquid media, and for DST, an additional 2–4 weeks or 7–10 days on solid or in liquid media, respectively (WHO, 2014).

Several tests have been introduced for rapid solid-based media DST. E-test relies on plastic strips impregnated with an exponential gradient of antibiotic concentrations to allow the direct determination of the minimum inhibitory concentration (MIC) based on the zone of inhibition on solid agar (Esteban et al., 2005). MDR/XDR-TB Color Test (TB-CX) is a single four-quadrant agar plate that detects Mtb growth in the clear zone and resistance to INH, RIF, and FQ [i.e., ciprofloxacin (CIP)] in yellow, green, and blue zones, respectively (Shibabaw et al., 2019).

Automated liquid-based culture systems such as BACTEC^TM^ MGIT^TM^ 960 automated system (Beckton Dickinson, Maryland, USA) (Scarparo et al., 2004), BacT/Alert® 3D system (bioMerieux, Durham, USA) (Angeby et al., 2003), and VersaTREK^TM^ system (Trek Diagnostic system, Ohio, USA) (Espasa et al., 2012) have been used for detection of first-line drug resistance, i.e., INH, RIF, streptomycin (STR), ethambutol (EMB), and pyrazinamide (PZA). These automated systems have growth indicators based on fluorescence signals, color changes, and pressure changes, respectively. Automated systems for second-line drug testing have been successfully implemented in diagnostic routines for MDR-TB patients (Gallo et al., 2017, Pinhata et al., 2024). Nonetheless, although automated systems have significantly reduced the turnaround time (TAT) for mycobacterial culture, their costs and the existence of alternative genetic and molecular methods for MDR-TB diagnosis, have preserved these techniques to be widely implemented in low-income countries (Parsons et al., 2011).

New innovative methods using microtiter plates have been developed to determine the MIC. For example, Sensititre® MycoTB (Trek Diagnostic system, Ohio, USA) uses a 96-well microtiter plate platform, where twelve drugs, including first-line (RIF, INH, STR, and EMB) and second-line drugs [OFL, MXF, Rifabutin (RFB), para-aminosalicylic acid (PAS), ethionamide (ETO), cycloserine (CYC), KAN, and AMK] at 8 different concentrations are lyophilized in individual wells and determining the resistance based on the presence of turbidity (Lee et al., 2014). However, the visual measurement of turbidity may underestimate the endpoint of bacterial growth. In this sense, the use of a colorimetric redox assay shows an advantage as it determines the growth based on color changes when an oxidation–reduction indicator is added to the medium (Rahman et al., 2004). Several redox indicators have been suggested to determine living and dead cells such as alamar blue (Cho et al., 2015), resazurin (Katawera et al., 2014), and 3-(4, 5-dimethylthiazol-2-yl)-2, 5- diphenyltetrazolium bromide (MTT) (Hundie et al., 2016). Both alamar blue and resazurin dyes are blue in an oxidized state and change to pink in a reduced state, indicating bacterial growth, while MTT is a yellow dye that turns purple (Cho et al., 2015, Katawera et al., 2014, Hundie et al., 2016). The *Comprehensive Research Prediction for Tuberculosis: an International Consortium* (CRyPTIC), has recently proposed cut-off values for 13 anti-tuberculosis drugs in a 96-well broth microdilution plate after collecting more than 20,000 clinical Mtb samples across the world (Consortium, 2022).

### 2.2 Genetic characterization of DR-TB

To address the global DR-TB crisis, one of the priority actions recommended by the WHO is to expand the rapid testing and detection of DR-TB cases (WHO, 2018). Generally, phenotypic testing has a long turnaround time to diagnose drug resistance and thus, rapid molecular assays have been developed to detect genetic mutations related to drug resistance and the results can be obtained in a few hours (Table 1). Some of these tests have been endorsed by WHO such as Xpert® Ultra MTB/RIF (Cepheid, Sunnyvale, USA), GenoType MTBDR (Hain Lifescience GmbH, Nehren, Germany), Truenat® MTB plus and Truenat®MTB tests (Truenat: Molbio Diagnostics, Bangalore, India), and Genoscholar™ NTM+MDRTB (Nipro Corporation, Osaka, Japan) (WHO, 2021).

**Table 1:** Commercialized kits for DR-TB.

Platform	Commercialized kit	Target genes related to drug resistance	Reference
Real-time polymerase chain reaction assay	BD MAX™ MDR-TB (Beckton Dickinson, Maryland, USA)	*rpoB* – RIF; *katG* and *inhA –* INH	(Ciesielczuk et al., 2020)
	Truenat™ MTB-RIF Dx (Molbio Diagnostics, Bangalore, India)	*rpoB* – RIF	(MacLean et al., 2020)
	Xpert® (Cepheid, Sunnyvale, USA)	MTB/RIF: *rpoB* – RIF MTB/XDR: *inhA, katG, fabG1,* and *oxyR-ahpC* – INH; *inhA –* ETO; *gyrA* and *gyrB –* FQ; *rrs* and *eis* – SLID	(Kebede et al., 2019, Y Cao et al., 2021)
	Abbott RealTime™ RIF/INH (Abbott Molecular, Illinois, USA)	*rpoB* – RIF; *katG* and *inhA –* INH	(Ruiz et al., 2017)
	Anyplex™ II (Seegene, Seoul, Korea)	MTB/MDR: *rpoB* – RIF; *katG* and *inhA –* INH MTB/XDR: *gyrA* – FQ; *rrs –* SLID	(Igarashi et al., 2017)
	Genedrive MTB/RIF ID Kit (Epistem, Manchester, United Kingdom)	*rpoB* – RIF	(Shenai et al., 2016)
	MeltPro® TB assay (Zeesan Biotech, Xiamen, China)	MTB/RIF: *rpoB* – RIF MTB/INH: *katG, inhA* and *oxyR-ahpC –* INH MTB/STR: *rpsL* – STR MTB/EMB: *embB -* EMB MTB/FQ: *gyrA -* FQ MTB/SL: *rrs* and *eis –* SLID	(Pang et al., 2016)
Line-probe assay (LiPA)	GenoType (Hain Lifescience GmbH, Nehren, Germany)	MTBDR*plus: rpoB* – RIF; *katG* and *inhA –* INH MTBDR*sl* version 1: *gyrA* – FQ; *rrs –* SLID; *embB –* EMB MTBDR*sl* version 2: *gyrA* and *gyrB* – FQ; *rrs* and *eis –* SLID	(Bai et al., 2016, Theron et al., 2016)
	Genoscholar™ (Nipro Corporation, Osaka, Japan)	NTM+MDRTB II: *rpoB* – RIF; *katG* and *inhA –* INH PZA TB II: *pncA* – PZA FQ+KM-TB II: *gyrA* – FQ; *rrs –* SLID	(Nathavitharana et al., 2016, Willby et al., 2018, Rigouts et al., 2019)
Microarray	VereMTB^TM^ Detection Kit (Veredus Laboratories, Singapore)	*rpoB* – RIF; *katG* and *inhA –* INH	(Ou et al., 2020)
	CapitalBio™ (CapitalBio Technology Inc., Beijing, China)	*rpoB* – RIF; *katG* and *inhA –* INH	(Zhang et al., 2018)
Liquid array system	FluoroType MTBDR (Hain Lifescience GmbH, Nehren, Germany)	*rpoB* – RIF; *katG* and *inhA –* INH	(Nguyen et al., 2019)

**Legend:** RIF: rifampicin; INH: isoniazid; ETO: ethionamide; FQ: fluoroquinolone; SLID: second-line injectable drug; STR: streptomycin; EMB: ethambutol; PZA: pyrazinamide.

Sputum viscosity, the presence of PCR inhibitors in sputum, and the difficulty to lyse the complex cell wall of Mtb (Kolia-Diafouka et al., 2018) have become the major issues in obtaining high-quality DNA for PCR amplification. Automated machines with integrated functions for sputum processing (liquefied, decontaminated, and/or deactivated), followed by DNA extraction, amplification, and detection are Xpert® MTB/RIF (Cao et al., 2021) and BD MAX™ MDR-TB (Beckton Dickinson, Maryland, USA) (Ciesielczuk et al., 2020). Alternatively, there are standalone automated DNA extraction systems such as Trueprep® AUTO (Molbio Diagnostics, India) (MacLean et al., 2020), Genolution Nextractor® NX-48 (Genolution Inc. Korea), Real-Prep system (Biosewoom, Seoul, Korea) (Kim et al., 2020), and TruTip® Automated Workstation (Akonni Biosystems, Maryland, USA) (Thakore et al., 2018) that could directly be used to extract nucleic acids from clinical specimens, i.e., sputum, bronchial washing, pericardial fluid, bronchial aspiration, and pleural fluid. For high-sensitivity DNA amplification and signal detection, most of the assays use multiplex-PCR with probe-based detection, either by real-time quantification (Ciesielczuk et al., 2020, MacLean et al., 2020, Kebede et al., 2019, Cao et al., 2021, Ruiz et al., 2017, Igarashi et al., 2017, Shenai et al., 2016, Pang et al., 2016), DNA-DNA hybridization in line-probe assays (LiPA) (Bai et al., 2016, Theron et al., 2016, Nathavitharana et al., 2016, Willby et al., 2018, Rigouts et al., 2019), chip-based detection in microarray (Ou et al., 2020, Zhang et al., 2018), or liquid array system (Nguyen et al., 2019) (Table 1).

These diagnostic kits were designed based on the most frequent single-nucleotide polymorphisms (SNPs) related to drug resistance. About 95% of RIF-resistant Mtb strains have mutations at codons 507 to 533 (81-bp) of *rpoB,* also known as the RIF Resistance Determinant Region (RRDR), commonly at codons 531, 513, and 526 (Ramaswamy and Musser, 1998). Among INH-resistant strains, 64% have a mutation at codon 315 of *katG*, 19% at position-15 of the *inhA* promoter region (*fabG1-inhA*), 1.2% at coding region of *inhA,* and 1.3% at *oxyR-ahpC* intergenic region (Seifert et al., 2015). In streptomycin (STR)-resistant strains, commonly reported mutations are in codons 43 and 88 of *rpsL,* 530 loop and 912 region of *rrs,* and *gidB* lineage marker (Shrestha et al., 2020). Mutations at the *embCAB* operon and *ubiA* gene are associated with ethambutol (EMB) resistance, especially at codons 306, 406, and 497 of *embB* (Xu et al., 2015). Out of 187 codons of the *pncA* gene, about 171 codons are responsible for pyrazinamide (PZA) resistance, especially at codons 3 to 17, 61 to 85, and 132 to 142 (Ramirez-Busby and Valafar, 2015). FQ-resistant strains most commonly have mutations at codons 74 to 113 of *gyrA*, and less commonly at codons 500 to 540 of *gyrB* (Pantel et al., 2012). For second-line injectable drug (SLID)-resistant strains, mutations in the16S rRNA gene *rrs* are related to AMK, CAP, and KAN resistance, especially at position A1401G, and mutations in the *eis* promoter are related to KAN resistance (Brossier et al., 2017).

Furthermore, these mutation points can serve as surrogate markers, e.g., 90% of RIF-resistant mutants are resistant to INH and thus, rifampicin resistance can be used as a marker to detect MDR-TB (Jaleta et al., 2017). Other reported surrogate markers that give additional prediction regarding drug resistance are PZA resistance which is associated with STR resistance (Xia et al., 2015) and *inhA* mutation showing cross-resistance to the INH-analogue drug, ethionamide (ETO) (Morlock et al., 2003).

Technically, the advantage of detecting these highly mutated points has also become a major concern for the correct identification of DR-TB as only one or few genes are included in the kits (Table 1) and mutations can occur outside of the predicted regions causing drug resistance (Nguyen et al., 2019). Thus, WHO has recommended the use of next-generation sequencing (NGS) technologies for multiple targeted genes or whole-genome sequencing (WGS) to detect drug resistance with higher genome coverage (WHO, 2023). Although WGS has provided a faster diagnosis for proper management of the patients, it could only predict about 82% of phenotypically DR-TB strains because of a lack of drug resistance profiles and the fact that some phenotypically drug-resistant isolates do not have any gene mutations (Wang et al., 2022). There is still a gap in the development of diagnostic tools and detection of DR-TB, and thus, having a database consisting of comprehensive mutation profiles of local strains is necessary for TB management. The WHO regularly updates a catalog of mutations in MTB and their association with drug resistance (WHO, 2023).

### 2.3 Characterization of DR-TB efflux pumps

Bacteria possess trans-membrane proteins spanning throughout their cell wall, also known as efflux pumps (EPs), that actively pump out substances such as toxics, waste, nutrients, and signaling molecules. They play a role in microbial resistance by expelling toxic substances, such as antibiotics, out of the cells. EP can be divided into six major families, i.e., ATP-binding cassette (ABC) transporter family, major facilitator superfamily (MFS), small multidrug resistance (SMR) family, resistance-nodulation-cell division (RND) family, multidrug and toxic compound extrusion (MATE) family, and PACE (proteobacterial antimicrobial compound efflux) (Paulsen et al., 1996, Kuroda and Tsuchiya, 2009, Moriyama et al., 2008). ABC transporters are primary transporters using energy generated from ATP hydrolysis, while the others are secondary transporters using proton motive force, such as H^+^ or Na^+^. Many studies have not only focused on the common genetic mutations related to drug resistance (as mentioned in Section 2.2), but also included the EPs in their investigations (Long et al., 2024). This is because some DR-TB strains do not have mutations in the commonly reported genes but mutations of EPs and their gene expression at basal level (without antibiotic stress) are higher compared to susceptible strains, suggesting its usefulness in the diagnosis of resistant TB (Li et al., 2015).

For example, many studies have shown the importance of Rv1258c (Tap), an MFS EP, in the detection of DR-TB. A total drug-resistant Mtb strain with a unique mutation at Rv1258c P369T was resistant to RIF, INH, STM, EMB, PZA, FQ, CAP, KAN, AMK, and ETO (Kanji et al., 2017). Another study showed that a clinical strain with a mutation at Rv1258c Y177H was resistant to AMK/KAN/CAP (Malinga et al., 2016). Also, multiple studies have strongly proved the importance of Rv1258c in resistance against RIF (Siddiqi et al., 2004), INH (Liu et al., 2019, Jiang et al., 2008), STR (Liu et al., 2019), PZA (Liu et al., 2019), FQ (Siddiqi et al., 2004), KAN (Malinga et al., 2016, Balganesh et al., 2012), and AMK (Malinga et al., 2016, Balganesh et al., 2012), among others, with at least 4-fold overexpression of Rv1258c or 4-fold changes in MICs.

Among the ABC EPs, Rv2936-Rv2937-Rv2938 (*drrA-drrB-drrC*) are the most common elements that were included in the detection of DR-TB. The *ddrA* and *ddrB* basal expression levels were significantly higher in MDR-TB and XDR-TB compared to a pan-sensitive group of strains (Li et al., 2015, Kanji et al., 2016). Under INH stress, DR-TB strains showed 4-fold overexpression of *ddrA* (Li et al., 2015), while under RIF stress, 4-fold overexpression of *drrB* and *drrC* were observed (Li et al., 2015). Moreover, a *M. smegmatis* plasmid construct carrying *drrAB*, increased the resistance to ethidium bromide (4-fold), daunorubicin (4-fold), ethambutol (8-fold), doxorubicin (3-fold), chloramphenicol (6-fold), erythromycin (4-fold), norfloxacin (4-fold), streptomycin (8-fold) and tetracycline (16-fold) (Choudhuri et al., 2002). Expression of the genes at basal levels is suitable for diagnostic purposes, while overexpression of the genes under drug exposure showed the importance of regulating drug resistance during treatment.

## Treatment of drug-resistant tuberculosis

For DR-TB, the specific regimen and duration of treatment depend on the extent and type of drug resistance. In 2016, WHO reclassified the anti-TB medicines or agents treatment regimen into four groups so that treatment regimen could be designed according to antimicrobial susceptibility to and tolerance: Group-A [levofloxacin (LXF), moxifloxacin (MFX), and gatifloxacin (GFX)]; Group-B [AMK, CAP, KAN, and (or STR)]; Group-C [ETO (or prothionamide, PTO), cycloserine (CYC) (or terizidone, TRD), linezolid (LZD), and clofazimine (CFZ)], and Group-D that was subclassified into three subgroups: D1 (PZA, EMB, and high-dose INH), D2 [bedaquiline (BDQ) and delamanid (DLM)], and D3 [p-aminosalicylic acid (PAS), imipenem–cilastatin (IPM-CLN), meropenem (MPM), and amoxicillin clavulanate (AMX/CLV) (or thioacetazone, TAZ) (WHO, 2016). These guidelines have been updated, but they hold to be important as for the first time, a standardized short-course regimen of 9–12 months was recommended for MDR-TB treatment instead of previous conventional regimens of over 18 months. In addition, the new classification of anti-TB drugs in groups paved the way for a dynamic definition of XDR-TB according to the updated regimens recommended by WHO. The current definition of XDR-TB is that TB caused by Mtb strains that in addition to RIF (and may also be resistant to INH) is also resistant to a FQ (LXF or MFX) and at least one additional Group-A drug (WHO, 2021). Therefore, any modification in Group-A drugs implies a change in the drugs included in the XDR-TB definition. Current WHO guidelines classify groups into Group-A (LFX or MXF; BDQ; LZD); Group-B (CYC; CFZ) and Group-C (EMB; DLM; PZA; IPM-CLN or MPM; AMK or STR; ETO or PTO; PAS) (WHO, 2022). The regimen for MDR/RR-TB patient includes all the three agents from Group-A and at least one agent from Group-B. If only one or two agents from Group-A are used, both Group-B agents have to be included and Group-C agents are added to complete the regimen. At least four drugs should be selected. Of note, a short-course regimen for MDR/RR-TB could be enterally oral (WHO, 2022).

In 2022, once results of PRACTECAL and Zenix studies were available (Conradie et al., 2022, Nyang'wa et al., 2024), WHO suggested a shorter 6-month treatment regimen of BDQ, pretomanid (PTM), LZD, with or without MFX (BPaL and BPaLM, respectively) in place of the shorter (9-month) or longer (18-month) regimen for MDR/RR-TB. BPaL regimen (without MFX) may be used for patients with documented resistance to FQ (WHO, 2022). Very recently WHO have updated recommendations for MDR-RR-TB treatment by including two new all oral regimens based on results obtained from the BEAT-Tuberculosis trial: 6-months regimen based on BDLLFxC (BDQ, DLM, LZD 600 mg, LXF, CFZ) and the EndTB trial: 9-month regimen comprising different combinations of BDQ, LXF or MFX, LZD, CFZ, DLM and PZA (WHO, 2024). In situations when the 6-month MDR/RR-TB regimen is not currently accessible, is not yet practicable to administer, or is not applicable to the patient, selected patients with MDR/RR-TB may benefit from a 9-month all-oral regimen comprising BDQ (6-month), in combination with LXF (or MFX), ETO (or LZD 2-month), EMB, high-dose INH, PZA, and CFZ (for 4-month, with the possibility of extending to 6-month, depending on sputum smear positivity), followed by treatment with LXF (or MFX), CFZ, EMB, and PZA (5-month) (WHO, 2022). Longer individualized regimens (18-month) as recommended by WHO in 2018 are available for the patients with MDR/RR-TB who are not eligible, intolerant, or fail in the 6-month or 9-month regimens, or with XDR-TB (WHO, 2022).

Despite the availability of treatment regimens for MDR/XDR-TB, the applicability of these therapies, especially in endemic regions, raises some concerns related to the cost incurred by the patients in already overwhelmed healthcare systems. Also, some drugs might not be always available to clinicians which results in treatment delay Moreover, compared to drug-susceptible TB, the treatment modalities for DR-TB require more extended treatment regimens, resulting in a lack of drug treatment compliance, incurring higher toxicity levels, and adverse effects (Migliori et al., 2020).

Alternatively, newer treatment modalities such as host-directed therapeutics (HDTs) could be utilized to improve treatment outcomes for DR-TB (Rao et al., 2019). These therapies modulate host inflammation and immunopathology to limit mycobacterial infection and pathology. Once an individual that is most likely to respond to HDTs is selected, generalized HDTs such as cytokine inhibitors (i.e., anti-IL-6 and anti-TNF-α) can be taken in the post-intensive phase of therapy, while vitamin supplementation (i.e., vitamin D and A), metformin, statins or non-steroidal anti-inflammatory drugs have been suggested as ancillary medication that can be taken concurrently with the anti-TB drugs (Kalra et al., 2023). However, the administration of these HDTs has not been validated so far.

Induction of mycobacterial efflux pumps may cause anti-TB drugs tolerance or even resistance (Adams et al., 2011). Consequently, efflux pump inhibitors (EPIs) would be an effective ancillary treatment for TB, including DR-TB (Li et al., 2015). Examples of EPIs are Ca^2+^ channel blockers [i.e., verapamil (VP), thioridazine (TZ), and chlorpromazine (CPZ)], protonophores [i.e., carbonyl cyanide m-chlorophenylhydrazone (CCCP), 2,4-dinitrophenol (DNP), and valinomycin (VAL)], and plant-derived EPIs [i.e., reserpine (RES), piperine (PIP), and berberine (BBR)], which significantly reduced the anti-TB drugs MICs of the studied isolates (Pule et al., 2016, Tegos et al., 2011) (Table 2). Significant effects of the EPIs depend on the EP system, for example, MFS can be inhibited by CCCP and DNP, and ABC transporters can be inhibited by VP in the presence of OFX resistance (Singh et al., 2011). As shown in Table 2, VP potentiates the effects of several anti-TB drugs such as RIF (Li et al., 2015), INH (Jaiswal et al., 2017, Rodrigues et al., 2012, Zhang et al., 2015), STR (Spies et al., 2008), EMB (Zhang et al., 2015, Srivastava et al., 2010), PZA (Zhang et al., 2017), FQ (Singh et al., 2011, Escribano et al., 2007), CAP (Machado et al., 2017), and BDQ (Xu et al., 2018) and this FDA-approved antihypertensive agent has been suggested as an adjunctive agent for TB treatment (Padmapriyadarsini et al., 2024).

**Table 2:** Synergistic effects of efflux pump inhibitors with anti-TB drugs.

Anti-TB drugs	EPI	Reference
RIF	VP, TZ, CPZ, CCP	(G Li et al., 2015)
INH	VP, CPZ, CCP, DNP, RES, tetrandrine	(Jaiswal et al., 2017, Rodrigues et al., 2012, Zhang et al., 2015)
STR	VP, CCCP	(Spies et al., 2008)
EMB	VP, RES, tetrandrine	(Zhang et al., 2015, Srivastava et al., 2010)
PZA	VP, RES, PIP	(Zhang et al., 2017)
FQ	VP, CCCP, DNP, RES, MC 207.110	(Singh et al., 2011, Escribano et al., 2007)
CAP	VP, TZ, CPZ	(Machado et al., 2017)
AMK	CPZ	(Machado et al., 2017)
BDQ	VP	(Xu et al., 2018)

**Legend:** RIF: rifampicin; INH: isoniazid; STR: streptomycin; EMB: ethambutol; PZA: pyrazinamide; FQ: fluoroquinolone; CAP: capreomycin; AMK: amikacin; BDQ: bedaquiline; VP: verapamil; TZ: thioridazine; CPZ: chlorpromazine; CCCP: carbonyl cyanide m-chlorophenylhydrazone; DNP: 2,4-dinitrophenol; RES: reserpine; PIP: piperine.

## Limitations of current diagnosis and treatment strategies against latent DR-TB

The risk of acquiring TBI after contact with an index TB patient depends on several factors, most importantly duration and closeness with the source TB case (Reichler et al., 2020, Praveen, 2020). Some underlying conditions such as human immunodeficiency virus (HIV) infection, end-stage renal disease, tobacco use, and diabetes mellitus may also contribute to the risk of becoming infected after exposure to Mtb (Njagi et al., 2023, Bandiara et al., 2022, Hu et al., 2024, Liu et al., 2022). Currently, there is no gold standard to diagnose TBI. The WHO recommended tuberculin skin test (TST) and interferon-gamma release assays (IGRAs) such as QuantiFERON®-TB Gold-In-Tube, QuantiFERON®-TB Gold Plus, QIAreach^TM^ QuantiFERON®-TB, T-SPOT®.TB, and Beijing Wantai's TB-IGRA for the detection of TBI (WHO, 2022). The diagnostic tests for TBI have limited efficacy, including false negative results in high-risk groups such as children, elderly, and immunocompromised patients, and have a poor positive predictive value to predict TB reactivation (Chin et al., 2023). Diagnosis of DR-TB infected individuals is far more challenging because it is an asymptomatic infection and there is a need to identify if the index patient is a DR-TB patient.

It was estimated that 19.1 million people are latently infected with MDR-TB (Knight et al., 2019). In many countries, MDR/XDR-TB patients face long delays before initiation of effective treatment and thus, close contacts are exposed to highly infectious MDR/XDR-TB patients for longer periods. Several studies that have addressed the risk of infection after exposure to a patient with MDR-TB, have demonstrated a (at least) similar risk of infection compared to patients exposed to DS-TB (Fox et al., 2017, Becerra et al., 2019, Krishnan et al., 2023). Moreover, a high risk of progression to MDR-TB disease has been recorded among household contacts of MDR/XDR-TB patients (Krishnan et al., 2023, Bamrah et al., 2014). Altogether, these studies suggest the need to implement systematic household contact investigations on who should be presumed to be infected with an MDR/XDR-TB strain until proven otherwise.

Three ongoing randomized trials address the efficacy of preventive treatment after exposure to an MDR-TB patient: namely TB-CHAMP (levofloxacin vs placebo); PHOENIx MDR-TB (delamanid vs INH) and VQUIN-MDR (levofloxacin vs placebo). At present, no results have been published. Nonetheless, data obtained from non-randomized, observational, or mathematical model studies, suggest the benefits of preventive treatment for MDR-TB contacts (Bamrah et al., 2014, Fox et al., 2015, Garcia-Prats et al., 2014, Dodd et al., 2022, Gureva et al., 2022, Apolisi et al., 2023). WHO guidelines recommend a 6-month preventive treatment with levofloxacin (WHO, 2020).

## Effectiveness of TB vaccines against DR-TB

Bacille Calmette–Guerin (BCG), an attenuated strain of *Mycobacterium bovis,* is the only vaccine available for TB since 1921. In 1924, BCG was distributed worldwide to multiple countries. The vaccine was maintained by subculturing until the more reliable and standardized seed-lot system was established, leading to its diversification into a number of genetically distinct substrains. Currently, BCG substrains can be divided into four major groups based on the tandem duplication-2 (DU2) forms: group I (BCG-Russia, BCG-Japan, and BCG-Moreau), group II (BCG-Sweden and BCG-Birkhaug), group III (BCG-Danish, BCG-Prague, BCG-Glaxo, and BCG-China), and group IV (BCG-Phipps, BCG-Tice, BCG-Frappier, and BCG-Pasteur) (Zhang et al., 2016, Bottai and Brosch, 2016). A study on 13 BCG strains in murine models showed that DU2 group IV exhibited the highest virulence, groups III and I moderate virulence, and group II the least virulence. Moreover, group IV was also more effective in providing protection against Mtb challenge (Zhang et al., 2016).

Furthermore, BCG vaccine effectiveness can be influenced by the host immune status. In immunocompromised patients such as human immunodeficiency virus (HIV)-infected infants, there is a high risk of disseminated BCG disease and severe impairment of BCG-specific T-cell responses (WHO, 2010). Also, exposure to environmental non-tuberculous mycobacteria (NTM) could resulted in variability in BCG protection. Mycobacterial species have a close genetic relationship and share various similar antigens. Therefore, exposure to NTM indirectly primed the host immune system and interferes with the generation of BCG-specific immunity, reducing the BCG vaccine efficacy (Ghasemi et al., 2024).

Although, people with historical BCG vaccination are more responsive to INH therapy (Prabowo et al., 2019), the BCG vaccine protective effects could be different in different Mtb subspecies (Kousha et al., 2021). In Brazil, it was reported that BCG vaccination provided 69% of protection in contacts against TB transmission from MDR-TB patients (Kritski et al., 1996). In contrast, it had been postulated that the BCG vaccine protects less efficiently against Beijing family strains, originating from China with a high virulence and probability to present MDR-TB genotypes. It was speculated that BCG mass vaccination in Southeast Asia has been a selective force for the emergence of the Beijing family genotypes and BCG vaccination could be a risk rather than protective against TB in populations infected by Beijing strains. Altogether BCG shows heterogeneous efficacy ranging from 0% to 80% (Lange et al., 2022).

Several new TB vaccines to prevent TB infection, disease, recurrence, and reinfection are in clinical trials to study their efficacy, safety, and immunogenicity (Zhuang et al., 2023). Most of the vaccines in trials have yet to be tested for their efficacy against DR-TB. Only Vaccae^TM^, a heat-killed *Mycobacterium vaccae* vaccine in a phase III clinical trial has shown promising results as adjunctive therapy in treating MDR-TB by improving sputum smear conversion from positive to negative, TB lesions, and TB cavitation (Huang and Hsieh, 2017). RUTI® vaccine is the only immunotherapeutic vaccine in phase II clinical trials that is developed for the treatment of TBI. RUTI® is composed of fragmented, purified, and liposomed heat-inactivated Mtb bacilli that are cultured under stress to induce latency antigens (Cardona, 2006). RUTI® increases the efficacy of anti-TB drugs, reduces the duration of treatment, and it shows a relatively acceptable safety profile (Nell et al., 2014). A clinical trial for RUTI® in MDR-TB patients was terminated due to a lack of patient recruitment (https://clinicaltrials.gov/ct2/show/NCT02711735). Other therapeutic vaccines proposed as adjunctive therapies are MIP (*Mycobacterium indicus pranii*, phase III), VPM1002 (recombinant BCG vaccine, phase III), H56:IC31 (phase I), ID93:GLA-SE (phase I), and TB-FLU-04L (phase IIa) (Li et al., 2017), but their efficacy for drug-resistant TB treatment has yet been tested (Parida et al., 2015). MTBVAC is the only attenuated TB vaccine (as BCG IS) under phase-III investigation in high TB incidence areas. MTBVAC contains genetic deletions in the genes phoP and fadD26 encoding two major virulence factors. It has already proven safe in infants, adolescents, and adults (Martín et al., 2021).

## Conclusion

Detection of MDR/XDR-TB could not be solely achieved with the common mutations used for the development of diagnostic kits. As such, molecular markers will always remain as a likely prediction of drug resistance and phenotypic DST may continue to play a relevant role as the gold standard. One of the aspects that could be further explored is the EPs, including screening on the expression of EPs in Mtb and determination of point mutations in EPs related to drug resistance. Furthermore, the advancement in next-generation sequencing helps to create a better database for genome-wide association study (GWAS) to determine the association between phenotypic resistance and genetic polymorphisms. Due to the difficulty in detecting a DR-TB patient and the low efficacy of therapeutic regimens against DR-TB, the close contacts with DR-TB patients have higher risk of developing active DR-TB. Moreover, the BCG vaccine's low protective effects, influenced by strain diversification, host immunity, NTM exposure, and the presence of Mtb Beijing strains (a major drug-resistant subgroup) minimize its potential role to block TB reactivation, contributing to generate the reservoir for transmission of DR-TB. Hence incorporating HDTs, EPIs, and/or immunotherapeutic vaccines complementary to available DR-TB treatments would be an ideal approach to prevent TB reactivation, limiting the growing number of DR-TB cases. To tackle the DR-TB crisis, it is of paramount importance to have better access and expand rapid molecular diagnosis to detect drug resistance and effective treatments. Judging from current control strategies against TB, the rise of MDR-TB and XDR-TB might further jeopardize the current efforts of TB control in the near future.

## CRediT authorship contribution statement

**Kai Ling Chin:** Conceptualization, Writing – original draft, Writing – review & editing. **Luis Anibarro:** Writing – review & editing. **Zi Yuan Chang:** Writing – review & editing. **Praneetha Palasuberniam:** Writing – review & editing. **Zainal Arifin Mustapha:** Writing – review & editing, Funding acquisition. **Maria E. Sarmiento:** Writing – review & editing. **Armando Acosta:** Writing – review & editing.

## Declaration of competing interest

The authors declare the following financial interests/personal relationships which may be considered as potential competing interests:

Zainal Arifin Mustapha reports financial support was provided by Malaysia Ministry of Higher Education. If there are other authors, they declare that they have no known competing financial interests or personal relationships that could have appeared to influence the work reported in this paper.

## Data Availability

No data was used for the research described in the article.
